# Analysis of Domestic and International Green Infrastructure Research Trends from the ESG Perspective in South Korea

**DOI:** 10.3390/ijerph19127099

**Published:** 2022-06-09

**Authors:** Eunjoung Lee, Gunwoo Kim

**Affiliations:** Graduate School of Urban Studies, Hanyang University, 222 Wangsimni-ro, Seongdong-gu, Seoul 04763, Korea; cocomo16@hanyang.ac.kr

**Keywords:** ESG, green infrastructure, sustainability, systematic review

## Abstract

Government-level ESG (environmental, social, and governance) institutionalization and active ESG activation in the private sector are being discussed for the first time this year in Korea, spurred by increased national interest since the COVID-19 pandemic crisis and the declaration of a carbon-neutral society by 2050, and ESG discussion in many fields is spreading rapidly. In addition, global awareness of the crisis caused by environmental pollution and natural disasters has highlighted the importance of green infrastructure (GI) as a new conceptual alternative to improve public value. Based on sustainability, which is a common goal of ESG and green infrastructure, this study aimed to examine the research targets and techniques of green infrastructure from the perspective of ESG. This study selected and analyzed 98 domestic and international academic journal papers published over the past 10 years in the Web of Science academic journal database literature collection. Focusing on the research subjects, the focus on green infrastructure, and research keywords, we examined the aspects of the green infrastructure plan that have been focused on from the ESG perspective and compared domestic and international research trends. In addition, implications for how each research topic is connected to the concept of ESG according to its function and purpose were derived. By examining the domestic and international research trends of green infrastructure from the ESG perspective, we identified the need for a wider range of research on the diversity and relationship between humans and the ecological environment; policies and systems; and technical research that does not focus only on a specific field. In this regard, we intend to increase the contribution to ESG management in the public sector through the establishment of green infrastructure plans and policies in the future, as they account for a large portion of public capital.

## 1. Introduction

ESG stands for “environment, social, and governance,” which are used as factors to judge a company’s non-financial performance, which is linked to corporate ethical or social responsibility investment [1,2], and this concept was presented in the UN Global Compact as a strategy for sustainable development.

Discussions on sustainability began in the 1990s, when the United Nations Conference on Environment and Development (UNCED) in Rio de Janeiro, Brazil, adopted Agenda 21, a plan for international cooperation for sustainable development. At this time, the world’s three major environmental conventions, the Convention on Climate Change, the Convention against Desertification, and the Convention on Biological Diversity, which are the basis of the environmental factors of ESG, were newly established.

Subsequently, the sustainable development goals (SDGs) presented by the UN at the 2015 UN Sustainable Development Summit were included in the 2030 Agenda for Sustainable Development as the 17 core sustainable development goals of the UN [3].

The 17 goals for sustainable development are ending poverty, ending hunger, health and welfare, education, gender equality, clean water hygiene, decent and clean energy, decent jobs and economic growth, industry and innovation and infrastructure, reducing inequality, sustainable cities and communities, responsible consumption and production, climate action, underwater ecosystems, peace and justice, and partnerships [4].

When the foundation of ESG was being laid, in 2011, Porter and Kreamer created the concept of Creating Shared Value (CSV), which seeks both social problem solving and economic value. It is a concept that suggests companies, consumers, and society should pursue shared values and form a complementary relationship through strategic social marketing beyond CSR [5].

ESG is an investment philosophy that pursues long-term value growth as the value of sustainable and harmonious development, considering the benefits of economic, environmental, social, and governance emphases [6].

The concept of ESG was first used in December 2004 in a report jointly produced by the United Nations and the Swiss Foreign Ministry, and details of environmental, social, and governance issues in “Who cars wins” are shown in Table 1 [7]. This is a concept applied to the corporate and investment sectors, but it is okay to apply it to the national, central, and urban governments with respect to ESG sector at this point in time [7].

As the concept of ESG becomes increasingly mainstream, ESG is widely considered, practiced, and popularized in the field of practical uses [6], and the areas involved in ESG are increasingly becoming more important.

Following the COVID-19 pandemic, sustainable and inclusive development has once again become a hot topic of discussion worldwide, and in response to increasingly serious sustainable development issues in environmental, social, and financial markets, international organizations and countries around the world have proposed sustainable and comprehensive development frameworks for human society [6].

As interest at the national level rises in Korea, along with the 2050 Declaration of Carbon Neutralization (10 December 2020), government-level ESG institutionalization and active ESG activation in the private sector are being discussed, and they have spread to many areas since they were first conceived a year ago.

The Korean government has required companies to disclose ESG obligations by 2025, and since declaring the Green New Deal policy and carbon neutrality, ESG has been promoted not only by the private sector but also by local governments (e.g., the city of Namyangju, using the slogan “Namyangju Runs Green”). Changes are taking place in the full-scale promotion of ESG administration [8], and ESG management, which was centered on private organizations and companies, is spreading to government agencies [9].

In addition, as part of this policy, green infrastructure—a conceptual alternative to improve urban environmental, cultural, and economic value—continues to be discussed in the domestic landscape field as a sustainable plan.

The first time the term “green infrastructure,” a combination of “green” and “infrastructure”, was used was in the policy report published by the Clinton administration in May 1999, entitled the President’s Council on Sustainable Development [10,11]. The policy report at the time presented “green infrastructure” as one of the five strategies for sustainable community development.

Green infrastructure creates a network of physical green spaces, the concept of greenways, to continuously utilize recreation functions in the city; it solves urban social problems by adding the concept of natural ecosystems to human ones [11].

Various benefits exist in green infrastructure [12], and the multifunctional benefits have the following three aspects:

First, contributing to the economic development of the community by improving the social, physical, and environmental conditions of the site [13].

Second, the promotion of leisure activities, realization of community’s aesthetic preference, improvement of residential environments through community regeneration and provision of comfort for human mental and physical health [14], and encouraging local residents’ voluntary participation in community environment management, granting socio-cultural benefits [15].

Third, ecological network contribution through biodiversity and habitat protection, environmental quality improvement, and ecological function of adaptation and mitigation of climate change [16].

The economic, socio-cultural, and ecological functions of green infrastructure become the basis for the introduction of green infrastructure in planning and land use by allowing them to be efficiently utilized in a limited space through the combination of functions [17]. As such, the establishment of a green infrastructure tailored to the local environment for sustainable development can be a key strategy for maintaining various environmental benefits and sustainable communities in the city.

In this study, to pursue sustainability, we examined the research trends of green infrastructure from an ESG perspective and examined the relationship between green infrastructure research subjects and items by ESG field. In the future, research on green infrastructure in Korea needs to include various studies related to this so that ESG management that pursues social, environmental, ethical, and sustainable growth as a policy tool in the public domain can be realized. Through this, it was judged that the evaluation factors for each ESG item as a strategy for creating a sustainable community environment from various developments can contribute to progressing the green infrastructure plan.

## 2. Materials and Methods

**Figure 1:**
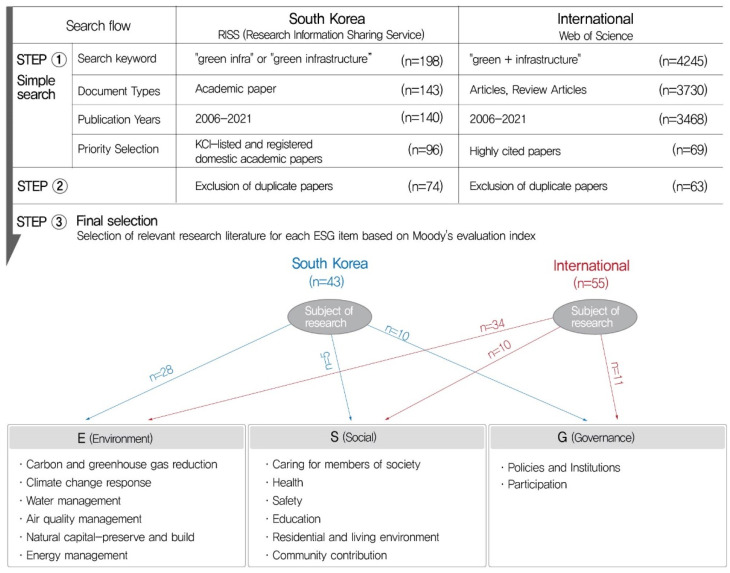
Research process.

This study used a systematic literature review [18]. Considering the characteristics of continuous research from the past to the present, the scope of research was limited to 2006–2021 to explore the “green infrastructure” trend and review the research subjects and fields in which the term is used. The specific research process is shown in Figure 1.

As a research method, papers from domestic and international academic journals were collected through an electronic journal database search.

The overseas database used for this study was the Highly Cited Paper Index in the Web of Science; domestic academic papers listed in the Korea Citation Index (KCI) and candidates for KCI registration were collected from RISS (the Research Information Sharing Service, http://www.riss.kr/, accessed on 5 March 2022) through a total of three stages of literature extraction.

The search keyword used for international search was “green + infrastructure” and the search keyword used for domestic search was “greeninfra” or “green infrastructure”.

In the first stage of the document selection process, in the simple search, keyword search (South Korea n = 198 articles, International n = 4245 articles), Document Type (South Korea n = 143 articles, International n = 3730 articles), and Publication Years, which were narrowed down to the period from 2006 to 2021 in Korea, when the study started, were set as parameters (South Korea n = 143, International n = 3730), and finally, academic papers that had secured public credibility by KIC listing in Korea and highly cited papers (South Korea n = 96, International n = 69) were selected.

The second stage was the exclusion of duplicate articles (74 domestic articles, 63 foreign articles), and in the third stage, after reviewing the keywords and abstracts of the searched articles, their thesis values, and effects, the classification by ESG items was pre-implemented based on the target of application.

To select studies meaningful for the analysis of the relationship between green infrastructure and ESG, first, Moody’s ESG evaluation index (https://www.moodys.com, accessed on 12 March 2022), one of the three major credit rating agencies in the United States, was used to select 55 international and 43 domestic research papers related to E (Environment), S (Social), and G (Governance) factors, with relevance for each category.

Among the 98 journal publications finally derived, the author reorganized and analyzed the publication status, research targets, and contents of each E, S, and G evaluation index based on major evaluation indicators being promoted overseas (international credit rating agencies) and domestic indicators (outside the Ministry of Trade, Industry, and Energy).

## 3. Results

### 3.1. ESG Evaluation Indicators

In line with the recent trend of emphasizing international environmental and social governance, ESG standards are being upgraded, and an appropriate foundation is being established. Although the evaluation indicators differ from institution to institution, the fundamental purpose they pursue is the same. Based on the major evaluation indicators being promoted overseas (international credit rating agencies) and domestic indicators (excluding the Ministry of Trade, Industry, and Energy), it was reorganized in Table 2 as follows.

### 3.2. Green Infrastructure Research Trends Related to ESG

#### 3.2.1. Status of Publication of Domestic and International Green Infrastructure Research Papers Related to ESG

**Figure 2:**
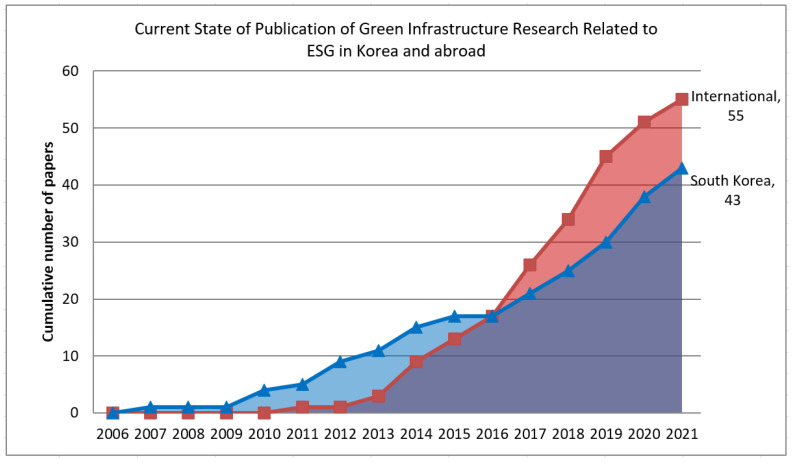
Current status of publication of domestic and international green infrastructure research related to ESG.

Analysis of the articles related to green infrastructure from 98 domestic and international journals showed that research has been actively conducted in Korea since 2007, and has been on a steep rise since 2016 (Figure 2).

Green infrastructure research, according to ESG items, was published with more than 60% of the focus on the environment both domestically and abroad, which is believed to be due to the early start of the concept of green infrastructure in the academic fields related to landscaping, environment, and civil engineering (Table 2). It can be seen that the remaining papers had a similar proportion in the order of governance and social factors, and research has recently expanded to various academic fields such as cities, architecture, and administration (Table 3).

#### 3.2.2. Green Infrastructure Research Trends According to ESG Evaluation Indicators

An analysis of the current status of research data published according to ESG evaluation indicators reconstructed by the author for domestic and international research trends showed that similar indicators were distributed in E (Environment) and G (Governance) items (Table 4).

The E (Environment) field showed a very similar research status in climate change response, with seven cases (16.3%) in Korea and nine cases (16.4%) overseas and in air quality management, with three cases (7.0%) in Korea and three cases (5.5%) overseas. Higher concentrations were found in water resource management (13 cases, 30.2%) and natural capital conservation and construction (5 cases, 11.6%) in Korea, and in water resource management (3 cases, 5.5%) and natural capital conservation and construction (19 cases, 34.5%) overseas.

In Korea, disaster management and water cycle research were treated as important subjects: damage from wind and water due to climate change accounts for more than 90% of all natural disasters, and it is concentrated in urban areas [23]. Research on green infrastructure for low-impact development techniques (LIDs) [24,25,26,27,28] in riparian areas, improvement of urban water circulation [29,30,31], and reduction in precipitation runoff [32,33,34,35] was conducted. Research on the preservation and construction of natural capital in Korea was conducted for green growth evaluation techniques [36,37], urban planning and GIS utilization planning techniques [38,39], and volume mapping research on vegetation sites [40]. In addition, studies were conducted to cope with climate change, focusing on topics such as the impact and utilization of green infrastructure on climate change [41,42,43], greenhouse gas reduction and thermal environment improvement [44,45,46,47], and air quality management such as fine dust reduction [48,49,50].

In foreign countries, research was actively conducted to secure green areas such as land and forests and to establish spaces for ecological diversity, and many studies were conducted on ecosystem services (ESs), ecosystem-based adaptations (EbAs), frameworks [15,51,52,53,54,55,56,57], and nature-based solutions (NbSs) [58,59,60,61], plans for securing green space and ecological diversity and policies [62,63,64,65,66], street trees, agricultural environment, and the effects provided by natural capital [67,68]. In addition, plans and models for adaptation to climate change [69,70,71,72,73], urban heat island mitigation [74,75,76,77], and green infrastructure utilization plans for air pollution reduction [78,79,80] were studied. In contrast to Korea, water resource management was partially studied in relation to water management for sustainability [81,82] and low-impact development [83].

However, research has not been conducted on energy management evaluation indicators related to renewable energy with active policies worldwide (Table 4).

In the S (Social) field, some studies related to housing and living environment (three cases, 7.0%) and community contribution (two cases, 4.7%) have been published in Korea, but studies related to health (seven cases, 12.7%) and consideration of social members (three cases, 5.5%) were conducted abroad.

In Korea, the main focus was on the level of user satisfaction [84,85,86] and the economic value [87,88] that green areas and parks can provide to residential and living environments. Overseas, research was conducted on human health and well-being [89,90,91,92], public health [93,94,95], and equity using green infrastructure [96,97,98] provided by green infrastructure.

However, there was no research on indicators in the education sector, such as safety, learning, and experience, for the effects of environmental damage or crime safety (Table 4).

The G (Government) field showed that studies on policies and systems (10 cases, or 23.3%, in Korea, and 12 cases, or 20.0%, abroad) were active in Korea and abroad, and that they had high expectations for urban and park development or continuation through green infrastructure.

In Korea, policy improvement of green infrastructure in connection with existing systems, such as urban planning, green park plans, land use plans, and urban-based projects [99,100,101,102], as well as research on evaluation techniques that support policies and systems [103,104,105,106] and certification plan studies [107], was conducted. In overseas countries, urban policies using green infrastructure [108,109,110], ESs, and other eco-logically based green city policies were implemented to study the effects of climate change and sustainability [111,112,113,114,115] and effects of joint policy efforts [116,117,118].

However, indicators related to participation in green infrastructure planning or policy establishment have not been studied (Table 4).

### 3.3. Domestic and International Green Infrastructure Research Trends and Implications

#### 3.3.1. Subjects and Contents of Domestic Green Infrastructure Research

The highest proportion of research on green infrastructure in Korea focused on large areas, such as cities and green areas, followed by units of green infrastructure such as parks, roads, green facilities, and waterfronts, which were studied in connection with the creation of an urban ecological environment and pollution reduction (Table 5).

From the ESG point of view, domestic green infrastructure research mainly focused on the G (Government) area, such as policies, plans, and evaluations for green infrastructure, compared to E (Environment) and S (Social) aspects.

The contents of urban policies and plans, evaluation of green infrastructure plans, and techniques of domestic green infrastructure research were actively studied. In particular, research measures for green infrastructure, green growth, and balanced development, such as urban planning and design techniques (five cases, 11.6%), LID (five cases, 11.6%), and green growth evaluation (five cases, 11.6%), were extensively handled, suggesting the importance of green infrastructure as a comprehensive and future policy at the city level (Table 6).

#### 3.3.2. International Green Infrastructure Research Subjects and Contents

Table 7 shows that, in Korea, internationally too, research on large-scale objects such as cities, green spaces, and green infrastructure accounted for a high proportion of green infrastructure research (Table 7).

In addition to policies, planning techniques, and evaluation, studies on urban biodiversity (16 cases, 29.1%), such as ESs, EbA measures and frameworks, and NbSs occupied the largest proportion.

In addition, various studies have been conducted to improve disaster management and the global environment, such as those focusing on green space and health and human well-being (six cases, 10.9%), urban heat islands (five cases, 9.1%), climate change (four cases, 7.3%), air quality (two cases, 3.6%), and water (three cases, 5.5%) (Table 8).

From an ESG point of view, various studies (S, E) were conducted on the relationship between humans’ health and the ecological environment, in addition to technological studies (G), such as policies and plans for green infrastructure, compared to Korea, which had no technological studies.

## 4. Discussion

### 4.1. Comparative Analysis of Domestic and International Green Infrastructure Research Trends

Comparing the published status of the analyzed green infrastructure research, the scale and expansion of research followed the order of E > G > S both in the domestic and international literature, but there were some differences in detailed ESG evaluation indicators. In the G field, only research on policies and systems was conducted in Korea and abroad, but in the E field, domestic water resources management was the main focus, while overseas, natural capital conservation and establishment were the most common subjects. In Korea, research on S aspects was conducted on housing, the living environment, and contribution to the local community, while overseas research was conducted on consideration of social members and health indicators.

In terms of green infrastructure research, Korea has also heavily focused on policies and plans for cities, parks, and green infrastructure, and some research on water resource management and vegetation environment was conducted. On the other hand, research on urban biodiversity was most active overseas, followed by planning and policies for cities, green infrastructure, and green areas, and research was conducted in various fields, such as urban heat islands, climate, air quality, water resources, and greening.

Korea focuses on short-term and practical utility, such as disaster and resource management, and improvement of people’s living and economic help. In foreign countries, the macroscopic relationship between humans and the ecological environment is important, and thus the focus is on securing ecological spaces both inside and outside the city, expanding social services, and living a healthy life.

### 4.2. Correlation between Green Infrastructure Research Subjects and Items by ESG Field

According to the conceptual definition and planning direction of green infrastructure, it is a physical space of various scales created for ecosystem services, recreational functions in the city, and solutions to urban social problems, where—in addition to space plans such as land use projects—integration and collaboration plans with public policies are also needed.

As a result of analyzing green infrastructure research, selected according to the evaluation index items of the ESG field reconstructed by the author, research activities in the E and G fields are expanding, but they are relatively low in the S field.

Research on policies, land use, and development techniques for both domestic and foreign cities accounted for the largest proportion of green infrastructure studies, and system studies for LID and urban ecological diversity were actively conducted.

This is considered as part of an effort to prepare an environmental response plan using green infrastructure targets of various scales, such as green areas, parks, roads, and green facilities, as global awareness of crisis is rising due to environmental pollution and disasters [72,113]. This means that green infrastructure, including ESs, is being actively accepted in the decision making in spatial planning and policy systems in cities to promote sustainable development [101,109].

### 4.3. The Development of Green Infrastructure Research from the ESG Perspective

For green infrastructure to contribute to ESG management in the public sector, sufficient research evidence and data are needed to support the validity of each field. As a result of analyzing the current status of green infrastructure research using the ESG evaluation index in this study, the fields that were relatively insufficient, or evaluation indicators that have not yet been studied, are as follows.

First, the subjects and contents of research that are currently biased toward the E and G fields should be further expanded to the S fields. It is clear that the implementation of green infrastructure plans or policies has a positive environmental and ecological impact. However, the social impact on human life requires long-term observation, and the amount of research and interest concerning this subject so far is not high. According to domestic and foreign research trends in the ESG evaluation index, research considering social members, health, housing and living environments, and community contribution is emerging, but there is no research on safety and education, and the transfer of research is insufficient compared to the E and G fields.

Second, for the policy use of green infrastructure, indicators related to green energy for carbon neutrality should be studied. In response to global warming, various efforts are being made to establish net-zero-carbon cities, rather than simply low-carbon cities, in line with global efforts to break away from the fossil fuel era to reduce greenhouse gas emissions. To prepare for it, fuel and renewable energy materials are required, together with urban spaces, industrial structures, and a green infrastructure environment [119]. In the E field, it is necessary to study the effects of green infrastructure on carbon reduction, greenhouse gas reduction, and energy management indicators.

Third, research on collaboration in green infrastructure plans or policies in the G field is needed. In ESG management, transparency, soundness, and value realization through communication of plans or policies are important, and must be accompanied by participation indicators. Since research on participation indicators has not yet been conducted at home or abroad, it seems necessary to study cases or measures to secure the reliability of policies through participation or communication in the planning or policy establishment stage of developing green infrastructure.

### 4.4. Development of Green Infrastructure Research in Korea

As the importance of green infrastructure for combatting climate change, disaster response, and sustainable growth has expanded, related research on the applicability and effectiveness of urban planning and policies is becoming more active, and Korea’s interest is similar to that found overseas.

However, according to the detailed evaluation indicators or research contents of each ESG field, Korea tends to concentrate on plans, techniques, and economic effects for disaster and resource management.

In the E field, research on natural capital conservation and construction indicators such as land and forests is passive, and research on urban ecological diversity has not yet been conducted.

In addition, in the S field, it seems to have focused only on short-term policy effects, focusing on residential, living environments, and community contribution indicators.

For Korean green infrastructure research to be consistent with the ESG management vision in the future, various considerations of the cycle of macroscopic human life and ecological environment, in addition to short-term effects and technology utilization, will be needed.

## 5. Conclusions

Although ESG and green infrastructure are commonly oriented toward sustainability and are actively embraced by national policies around the world, no research or consideration has been dedicated to the relationship between green infrastructure and the ESG perspective.

To analyze the relationship between green infrastructure and ESG, 55 research papers from abroad and 43 from Korea that meet the ESG evaluation index criteria were selected and considered among green infrastructure studies from 2006 to 2021. The selected papers analyzed the status of green infrastructure research related to ESG, green infrastructure research trends according to the ESG evaluation index, and green infrastructure research subjects and contents, respectively, and the author reconstructed and used the ESG evaluation index based on detailed evaluation items developed by domestic and foreign public and private evaluation institutions.

As a result, research on green infrastructure in both Korea and abroad is expanding in the E, S, and G fields, and various studies are also distributed in the detailed ESG evaluation index reconstructed by the author. This means that the green infrastructure is largely in line with the goals and indicators of ESG management, and suggests that the establishment of green infrastructure will contribute to ESG management in the public sector in the future.

This study reveals that green infrastructure is a useful environmental, social, and policy tool for realizing ESG management in pursuit of social, environmental, and ethical sustainable growth in the public domain, and it can be used to present ESG evaluation indicators in future green infrastructure plans and policies.

To this end, the direction of future green infrastructure research is presented as follows.

First, for green infrastructure to contribute to ESG management in the public sector, sufficient research evidence and data are needed to support the validity of each field, E, S, and G, so research in the S field, which is relatively insufficient compared to the E and G fields, should be expanded.

Second, research in fields lacking in literature according to the ESG evaluation index is needed. As the E field’s concern with carbon reduction, greenhouse gas reduction, and energy management, the S field’s focus on safety and education, and the G field’s participation indicators are increasingly important in public ESG management globally, related research will be essential in the future.

Third, green infrastructure plans and policies account for a large portion of public capital, and since most of the infrastructure projects are carried out as long-term projects, sufficient validity will be provided when research on human and ecological environment diversity and relationships is supported from a macro perspective. As Korea focuses on research on short-term effects and technical aspects, studies on macroscopic human life and ecological environment cycles are essential.

## Figures and Tables

**Table 1:** ESG itemized initial concepts.

Category	Detail Item	Key Points
Environment issues	Risks associated with climate change; Efforts to reduce environmental pollution and waste; Strengthen environmental regulations on products and services; Risk of reputation of civil society for performance, transparency, and responsible management; Responding to new markets for environmental services and green products.	Performance, Transparency, Accountability
Social issues	Workplace safety and health; Local communities and relationships; Respect human rights in contracts with suppliers and partners; Government and local community relationships in developing countries; Risk of reputation of civil society for performance, transparency, and responsible management.	Community relations
Governance issues	Board structure and accountability; Accounting transparency; Structure of audit committee and independence of audit; Managerial compensation; Management of corruption and bribery.	Transparency management

Source: UNGC (UN Global Compact) and Swiss Federal Department of Foreign Affairs, (2004:12).

**Table 2:** Domestic and foreign ESG evaluation indicators.

Category	ESG Evaluation Indicators (Reorganized by Author to Analyze Research Trends in Green Infrastructure at Home and Abroad)	Moody’s (National ESG Evaluation Report) [19]	K-ESG (Ministry of Trade, Industry, and Energy) Guideline’s Key Items [20]	ESG Promotion Strategy for Domestic Public Institutions (Korea Rural Community Corporation) [21]	Domestic Research Results of ESG Evaluation Indicators for Public Institutions [22]
E (Environment)	Carbon reduction and greenhouse gas reduction	Carbon conversion	Greenhouse gas emissions	Reaching carbon neutrality by expanding renewable energy	Climate change and greenhouse gas reduction
	Combating climate change (reducing urban heat island effects, etc.)	Climate change	Ratio of energy and renewable energy use	Green energy	Environmental efforts
	Water resource management (reduction in flood and runoff, disaster, water, water quality protection, etc.)	Water resource management	Waste discharge	Carbon reduction	Eco-friendly products and services
	Air quality management (air pollution improvement, air purification, etc.)	Waste and pollution	Air pollutant emissions	Construction of a green ecosystem	Safety and risk management
	Conservation and construction of natural capital (land, forest, ecological diversity, etc.)	Natural capital (land, forests, ecological diversity, etc.)	Water pollutant emissions		
	Energy management		Certified eco-friendly products and services		
S (Social)	Consideration of Members of Society (Providing Opportunities for Service Benefits, etc.)	Population	Socially Responsible Management Policy	A safe and Happy Life	Job Creation
	Health (health promotion, etc.)	Labor and income	Formulation of human rights policies and risk assessment	Creation of hope	Human rights/labor practices
	Safety (environmental damage, crime safety, etc.)	Health and safety	Safety and health promotion system and industrial accident rate	Health and vigor	Ethical management
	Education (learning, experience, etc.)	Education	Strategic social contribution (community contribution)	Security guard	Fairness, protection of the weak, and social integration
	Residential and living environment (improvement of comfort, etc.)	Residence	Compliance with social laws/regulations		Community participation and development
	Community contributions (regional economy and tourism revitalization, etc.)	Basic service accessibility	Information protection		
G (Governance)	Policies and Systems	Institutional Structure	Board Diversity, Activities, etc.	Realizing Common Values Together with the People	Leadership
	Participation	Policy reliability and effectiveness	Board structure	Participation	Board of directors operation
		Transparency and information disclosure	Ethical management	Transparency	Transparent management
		Budget management	Compliance with auditing and governance regulations	Integrity	Fiscal soundness
					Stakeholder communication
					Compliance with policy

Source: author, based on detailed evaluation items by domestic and international public and private evaluation institutions.

**Table 3:** Domestic and foreign ESG evaluation indicators.

Category		Total/f (%)	2006–2010	2011–2015	2016–2021
**Korea**	**E (Environment)**	28	1	11	16
		(65.1)	(2.3)	(25.6)	(37.2)
	**S (Social)**	5	2	-	3
		(11.6)	(4.7)		(7)
	**G (Governance)**	10	1	2	7
		(23.3)	(2.3)	(4.7)	(16.3)
	Total	43	4	13	26
		(100)	(9.3)	(30.2)	(60.5)
**Overseas**	**E (Environment)**	34	-	10	24
		(61.8)		(18.2)	(43.6)
	**S (Social)**	10	-	2	8
		(18.2)		(3.6)	(14.5)
	**G (Governance)**	11	-	1	10
		(20)		(1.8)	(18.2)
	Total	55	-	13	42
		(100)		(23.6)	(76.4)

**Table 4:** Status of publication of domestic and international green infrastructure research according to ESG evaluation index.

Category	ESG Evaluation Indicators (Reorganized by Author to Analyze Research Trends in Green Infrastructure at Home and Abroad)	South Korea		Overseas Country	
		Papers	%	Papers	%
**E (Environment)**	Carbon and greenhouse gas reduction	0	-	0	-
	Climate change response (Reduction in urban heat island effect, etc.)	7	16.3	9	16.4
	Water management (flood and runoff reduction, disaster, water, water quality protection, etc.)	13	30.2	3	5.5
	Air quality management (Improvement of air pollution, air purification, etc.)	3	7	3	5.5
	Natural capital—preserve and build (land, forests, ecological diversity, etc.)	5	11.6	19	34.5
	Energy management	0	-	0	-
	Subtotal	28	65.1	34	61.8
**S (Social)**	Caring for members of society	0	-	3	5.5
	(Service benefit, opportunities, etc.)				
	Health (health promotion, etc.)	0	-	7	12.7
	Safety (environmental damage, crime safety, etc.)	0	-	0	-
	Education (learning, experience, etc.)	0	-	0	-
	Residential and living environment (improvement of comfort, etc.)	3	7	0	-
	Community contribution (revitalizing the local economy and tourism)	2	4.7	0	-
	Subtotal	5	11.6	10	18.2
**G (Governance)**	Policies and institutions	10	23.3	11	20
	Participation	0	-	0	-
	Subtotal	10	23.3	11	20
	Total	43	100	55	100

**Table 5:** Domestic green infrastructure research target in South Korea.

Subject	Detail Subject	Total	F (%)
Cities	Urban policy and development, land use, neighborhood complexes, housing development sites, etc.	14	32.6
Green spaces	Urban forests, ecological green axis, vegetation settlement, development-restricted area, urban base green areas, non-urbanized land, green buffer areas, etc.	7	16.3
Green infrastructure	-	-	-
Parks	Urban parks, etc.	6	14
Road	Roads, highways, greenways, etc.	5	11.6
Greening	Wall greening, green walls, green infrastructure	4	9.4
Ecosystem	-	-	-
Water	Waterfront and hydrophilic spaces, Flood-prone areas, non-point sources, etc.	5	11.6
Air-contaminated area	Vulnerable areas of fine dust	2	4.7
Total		43	100

**Table 6:** Domestic green infrastructure research content in South Korea.

Category	Research Content	Total	F (%)
Urban policies and plans	Evaluation and improvement of legal systems and policies	3	7.0
	Urban planning and design techniques	5	11.6
	LID	5	11.6
	Environmentally friendly complex certification (LEED-ND)	2	4.7
	Evaluation of land use characteristics and urban resilience	2	4.7
	GIS utilization techniques	1	2.3
	Subtotal	18	41.9
Green spaces	Park policies and plans	3	7
	Subtotal	3	7
Green infrastructure	Green growth assessment	5	9.3
	Green infrastructure planning technique	4	11.6
	Subtotal	9	9.3
Urban biodiversity	Vegetation settlement, vegetation volume mapping study	2	4.7
	Ecological impact assessment	1	2.3
	Vegetation (wall, road, etc.)	3	7
	Subtotal	6	14
Water resources and disaster management	Improvement of excellent management techniques and water circulation	4	9.3
	Disaster prevention function and atmospheric management evaluation	3	7.0
	Subtotal	7	16.3
Total		43	100

**Table 7:** International green infrastructure research target.

Subject	Detail Subject	Total	F (%)
Cities	Cities, land use, urban development, urban environment, urban planning, urban vacancy, and land use legacies	19	34.5
Green spaces	Green spaces, agricultural land, neighborhood greenness, urban forest, urban green spaces (UGS), urban green–blue spaces, urban nature	13	23.6
Green infrastructure	Urban green infrastructure	9	16.4
Parks	Parks, urban gardens, urban green parks	4	7.3
Road	Roads, streets	2	3.6
Greening	Urban greening, vegetation barriers	3	5.5
Ecosystem	Ecosystem, NbSs	3	5.5
Water	Water management, stormwater management	2	3.6
Air-contaminated area	-	-	-
Total		55	100

**Table 8:** International green infrastructure research.

Category	Research Content	Total	F (%)
Urban policy and planning	Urban planning valuation and framework, socioecological system, urban resilience study	6	10.9
	Policy options for managing urban growth	1	1.8
	Subtotal	7	12.7
Green spaces	Green space and health, human well-being	6	10.9
	Green space networks, framework, quantifying, planning	4	7.3
	Agri-environmental schemes (AESs)	1	1.8
	Applying GSM data	1	1.8
	Subtotal	12	21.8
Green Infrastructure	Green infrastructure planning and technologies	4	7.3
	Subtotal	4	7.3
Urban biodiversity	Ecosystem services (ESs), ecosystem-based adaptation (EbA) measures and frameworks	11	20
	Nature-based solutions (NbSs)	5	9.1
	Designing vegetation barriers, urban planting	2	3.6
	Subtotal	18	32.7
Water resources and disaster management	Reducing urban heat stress, cooling the environment	5	9.1
	Climate change adaptation and mitigation plans and model	4	7.3
	Improving urban air quality	2	3.6
	Water management, stormwater control, techniques	3	5.5
	Subtotal	14	25.5
Total		55	100

## Data Availability

The data presented in this study are available on request from the corresponding author.
